# Spatial and temporal patterns of gene expression during neurogenesis in the sea urchin *Lytechinus variegatus*

**DOI:** 10.1186/s13227-019-0115-8

**Published:** 2019-02-12

**Authors:** Leslie A. Slota, Esther M. Miranda, David R. McClay

**Affiliations:** Department of Biology, Duke University, 124 Science Dr., Box 90338, Durham, NC 27708 USA

**Keywords:** Neurogenesis, Neural specification, Sea urchin, Gene regulatory state

## Abstract

**Background:**

The sea urchin is a basal deuterostome that is more closely related to vertebrates than many organisms traditionally used to study neurogenesis. This phylogenetic position means that the sea urchin can provide insights into the evolution of the nervous system by helping resolve which developmental processes are deuterostome innovations, which are innovations in other clades, and which are ancestral. However, the nervous system of echinoderms is one of the least understood of all major metazoan phyla. To gain insights into echinoderm neurogenesis, spatial and temporal gene expression data are essential. Then, functional data will enable the building of a detailed gene regulatory network for neurogenesis in the sea urchin that can be compared across metazoans to resolve questions about how nervous systems evolved.

**Results:**

Here, we analyze spatiotemporal gene expression during sea urchin neurogenesis for genes that have been shown to be neurogenic in one or more species. We report the expression of 21 genes expressed in areas of neurogenesis in the sea urchin embryo from blastula stage (just before neural progenitors begin their specification sequence) through pluteus larval stage (when much of the nervous system has been patterned). Among those 21 gene expression patterns, we report expression of 11 transcription factors and 2 axon guidance genes, each expressed in discrete domains in the neuroectoderm or in the endoderm. Most of these genes are expressed in and around the ciliary band. Some including the transcription factors *Lv*-*mbx, Lv*-*dmrt, Lv*-*islet,* and *Lv*-*atbf1,* the nuclear protein *Lv*-*prohibitin*, and the guidance molecule *Lv*-*semaa* are expressed in the endoderm where they are presumably involved in neurogenesis in the gut.

**Conclusions:**

This study builds a foundation to study how neurons are specified and evolved by analyzing spatial and temporal gene expression during neurogenesis in a basal deuterostome. With these expression patterns, we will be able to understand what genes are required for neural development in the sea urchin. These data can be used as a starting point to (1) build a spatial gene regulatory network for sea urchin neurogenesis, (2) identify how subtypes of neurons are specified, (3) perform comparative studies with the sea urchin, protostome, and vertebrate organisms.

**Electronic supplementary material:**

The online version of this article (10.1186/s13227-019-0115-8) contains supplementary material, which is available to authorized users.

## Background

The formation of a nervous system is a key innovation in evolution that allows for an organism to integrate sensory information and interact with its environment through motor output. Neurogenesis is a tightly controlled process that occurs during embryonic development in both indirect (animals with larval stages) and direct developing organisms. Comparisons of genomic sequences across the animal kingdom indicate that metazoan nervous systems largely employ a conserved set of genes during embryonic development [1]. Studying those genes in a number of organisms, especially those with simplified nervous systems, has the promise of revealing not only how nervous systems develop, but also how they evolved. Over the past decade, much has been learned about the origins of the vertebrate nervous system, and information has accumulated for neurogenesis of other deuterostomes, including the sea urchin. Nevertheless, much remains to be learned about how complex nervous systems develop.

Echinoderms are a basal deuterostome group characterized by pentaradial symmetry. Unlike the adults, echinoderm embryos are bilaterally symmetric. The nervous system of the bilaterally symmetric sea urchin embryo is composed of 3 major regions. The first is the apical organ, which is a collection of 2–6 serotonergic neurons as well as several other neural cell types found in the anterior end of the embryo [2–4]. The apical organ is considered to be the embryonic central nervous system and the processing center of the embryo [5]. The second region is the ciliary band, an ectodermal band of ciliated cells and neurons thought to aid in swimming and sensory behaviors [6–8]. Associated with the ciliary band is a collection of neurons known as the postoral neurons which lie just outside the ciliary band and connect axons to the ciliary band axonal tract [9]. Together, the ciliary band and postoral neurons are considered to be part of the peripheral nervous system of the embryo [5, 9]. The third region of the embryonic nervous system is in the endoderm where several neurons within the gut, as well as in the developing mouth and anus, aid in swallowing and movement of food through the digestive tract. Neurons in the gut have been shown to be specified directly within the endoderm [10].

Over the last 20 years, much effort has been put into creating a developmental gene regulatory network (GRN) for sea urchin development. A big advance in that research occurred with publication of the first sea urchin genome in 2006, and the availability of the genomic information spurred deeper insights into many properties of the embryo [11]. During the annotation phase of the genome project, a large number of dedicated neural molecules were identified [12], and over the last several years, efforts have begun to build GRN states for the sea urchin [2, 5, 13, 14]. However, only a limited number of these neural molecules have been spatiotemporally and functionally characterized. Fewer than 20 specifiers have been localized to neural precursors and assembled into GRN models of the more than 70 transcription factors identified in the genome (based on sequence orthologs of transcription factors identified as neural in other systems) [2, 5, 12–15].

Expanding the sea urchin neurogenic GRN can be beneficial for many reasons. The first is the evolutionary insights that can be gained. The phylogenetic position of sea urchins as a basal deuterostome has already shown, with a limited dataset, that sea urchin neurogenesis resembles vertebrate neurogenesis, more than protostome neurogenesis [2]. This suggests that deuterostome neurogenesis is quite conserved. An examination of how sea urchin neurogenesis occurs can be extremely informative, particularly in instances where neurogenic GRNs differ between deuterostomes and protostomes. Another compelling reason for exploring sea urchin neurogenesis is from a developmental perspective; the simplicity and speed of neurogenesis in the sea urchin embryo, along with an ability to identify and place GRN components efficiently, allow for a thorough dissection of which genes are used and how they are used to build a deuterostome nervous system.

The transcription factors shown to be necessary for neurogenesis in the sea urchin include *soxC* and *brn1/2/4*, which are expressed broadly in the nervous system and are required for neurons throughout the embryo [5, 13]. Furthermore, some transcription factors are expressed only by specific types of neural progenitors in the sea urchin and are required for the development of subsets of neurons in the nervous system. *Neurogenin* is required for specification of cholinergic neurons in the ciliary band, *orthopedia* is required for specification of postoral neurons, and *achaete*–*scute* and *zfhx1* have been shown to be required for specification or differentiation of serotonergic neurons in the apical organ, respectively [2, 13, 15]. Additionally, *soxb1, insm,* and *sip1* are expressed during the initial proneural specification period in the sea urchin [14]. Perturbations of these transcription factors led to the construction of preliminary gene regulatory networks for neurogenesis. Inhibition of signaling pathways such as Nodal, FGF, Delta/Notch and signaling molecules such as Wnt ligands has also contributed to the knowledge of inputs into nervous system development in the sea urchin [2, 5, 14, 15]. Before GRNs can be assembled to reveal detailed neurogenic specification sequences, and allow for evolutionary comparisons, many additional neural transcription factors and signaling molecules must be characterized. Thus, identification of additional genes involved in neurogenesis and their spatiotemporal expression profiles is a necessary beginning point toward that goal. With a vastly expanded group of involved genes, one will then be able to ask questions about patterning: Is neural development similar to vertebrates? Are the same genes used in similar ways as flies and vertebrates? Which neural processes and phenomena are vertebrate or deuterostome innovations, and which evolved earlier? What are the innovations (if any) made within the echinoderm lineage to get their unique organization of the nervous system?

Here we report the spatial and temporal expression patterns of 21 genes from blastula through pluteus stage in the sea urchin *Lytechinus variegatus* that are expressed in areas of the nervous system and have been reported to be involved in neurogenesis in other model systems. These patterns of gene expression provide a template for future perturbation studies to determine whether they are involved in, or essential for, proper neural patterning and behavior in the sea urchin and ultimately for understanding how neural GRNs are established in development. However, these patterns of gene expression provide more than a simple list of molecular players for sea urchin neurogenesis. The data presented here reveal the incredibly dynamic nature of gene expression required for neurogenesis to occur. Some of these expression patterns are complex and show the initial expression in a non-neural area of the embryo early, then turn off, and later express in areas of the nervous system. Others come on later in developmental time, with their expression remaining in a single area of the nervous system. Still others are expressed in multiple neurogenic areas previously shown to have unique regulatory states [2, 14].

Taken together, the data presented here reveal that even simple embryos, such as the sea urchin, must possess complex gene regulatory networks to build a functioning nervous system. We believe that the sea urchin embryo—with its known developmental gene regulatory network, molecular tractability, and simple nervous system—can be a developmental model for understanding of how a nervous system is built. Indeed, the sea urchin is unique in that it is one of the few organisms that have the potential for revealing gene regulatory networks, beginning from the initial maternal inputs through zygotic transcription, that lead to a functioning nervous system. To this end, the data shown here are a critical and necessary step to bring the sea urchin to the forefront of neural development research.

## Results

### Identification of genes expressed in the embryonic sea urchin nervous system

As part of an effort to identify components of the neurogenic GRN in the sea urchin, a molecular cloning and an in situ hybridization screening were performed. To choose candidate genes for this screening, we queried the published *Stronglyocentrotus purpuratus* developmental transcriptome (www.echinobase.org) and focused on annotated sea urchin orthologs of genes expressed during embryonic development that belong to the functional classification of neurogenesis [16]. For each candidate chosen, coding sequences were isolated from *Lytechinus variegatus* cDNA and spatiotemporal expression was analyzed by in situ hybridization from blastula stage through pluteus larval stage. These time points were chosen so that the in situ screening covered hatched blastula stage, the onset of earliest neural specification, through pluteus stage, when much of the nervous system has been patterned and larvae have begun to use their simple nervous system. Within this time frame, embryos were fixed every 2 h in development and candidate genes were examined so that dynamically changing expression of these genes and their spatial distribution could be detected.

### Transcription factors expressed in the apical organ

**Fig. 1:**
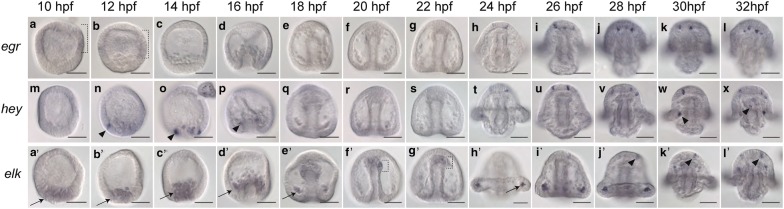
Transcription factors expressed in the apical organ. **a**–**l** *Lv*-*egr* is expressed in the anterior two-thirds of the embryo from 10 to 12 hpf (hours post fertilization) (brackets in **a**–**b**) and then comes on in cells in the apical organ starting at 24 hpf (**h**). **m**–**x** *Lv*-*hey* is expressed in scattered cells of the vegetal plate from 10 to 12 hpf (arrowheads in **n**, **o**). Inset image of **o** shows posterior view of vegetal plate. One to two cells express *Lv*-*hey* in the elongating archenteron at 16 hpf (arrowhead in **p**). Then, at 24 hpf, *Lv*-*hey* is expressed in 2–4 cells in the apical organ (**t**–**x**). *Lv*-*hey* is also expressed in scattered mesodermal cells at 30 hpf onward (arrowhead in W) and in 1–2 cells in the gut endoderm (arrowhead in **x**). **a′**–**l′** *Lv*-*elk* is expressed lightly in skeletogenic mesodermal cells from 10 to 18 hpf (**a′**–**e′**) (arrows). From 20 to 22 hpf, *Lv*-*elk* is expressed in the non-skeletogenic mesoderm (brackets in **f′**, **g′**). At 24 hpf, *Lv*-*elk* turns off in the non-skeletogenic mesoderm and turns back on in a subset of skeletogenic mesoderm (arrow in H′). At 28 hpf, *Lv*-*elk* is expressed in 1–2 cells of the apical organ (arrowheads in **j′**–**l′**). Scale bars = 50 μm

Members of the Early Growth Factor Response (Egr) family are zinc finger transcription factors and include the vertebrate gene *krox20* which is involved in the development of the hindbrain and neural crest [17, 18]. *Lv*-*egr* is detected very faintly at 10–12 h postfertilization (hpf) in the anterior two-thirds of the embryo (brackets in Fig. 1a, b). This expression is then diminished (Fig. 1c–g), and *Lv*-*egr* is expressed in cells of the apical organ starting at 24 hpf (Fig. 1h–l). In many pluteus-stage embryos, *Lv*-*egr* is strongly expressed in a single cell, while 1–4 other cells faintly express *Lv*-*egr* in the apical organ. The beginning of the specification sequence of neural progenitors in the apical organ, which is marked by expression of *soxC* and *delta*, has been shown to begin at blastula stages [2, 5]. Since expression of *Lv*-*egr* in the apical organ does not begin until 24 hpf (early pluteus stage), which is several hours after the onset of *delta* and *soxc*, this suggests that *Lv*-*egr* is most likely involved in differentiation or survival of neurons there rather than in specification of progenitors.

Hey (hairy/enhancer of split related with YRPW motif) proteins are basic helix–loop–helix transcription factors that are targets of Notch signaling. In vertebrates, *hey* genes have many different effects on the developing nervous system including, but not limited to: positively regulating maintenance of neural precursors, antagonism of some neural subtypes, and promotion of neural differentiation [19–22]. The *Drosophila hey* otholog is expressed by differentiating neurons and is involved in asymmetric division of ganglion mother cells [20]. *Lv*-*hey* is first expressed at mesenchyme blastula stages (12–14 hpf) in a salt and pepper ring of cells in the vegetal plate (Fig. 1m, arrowheads in Fig. 1n–o). This expression in the vegetal plate is most likely mesodermal. As gastrulation begins, *Lv*-*hey* is expressed lightly in 2 cells on either side of the archenteron (Fig. 1p arrowhead). *Lv*-*hey* then cannot be detected by in situ hybridization until 24 hpf when it turns on in 2–4 cells in the apical organ (Fig. 1t). *Lv*-*hey* remains in 2–4 cells in the apical organ and by 30 hpf is also expressed in 1–2 cells at the base of the larval arms, which are likely mesodermal (arrowhead in Fig. 1w). At this time point, some embryos have *Lv*-*hey* expression in several cells in the gut, but that is not consistent in all embryos (arrowhead in Fig. 1x). The late onset of expression in the apical organ suggests that, like *Lv*-*egr, Lv*-*hey* is likely not involved specification of neural progenitors. Rather, it is possible that *Lv*-*hey* is used in the maintenance of neural precursors or in the differentiation of neurons, like the role in of *hey* genes in vertebrate models.

Elk, an Ets family transcription factor, has been shown be expressed in the rodent brain [23, 24]. Knockdown studies have shown that Elk-1 effects transcription of several genes in the mouse brain and is considered a pro-differentiation factor there [25]. In the sea urchin *S. purpuratus*, *elk* has been shown to suppress apoptosis and promote cell proliferation in the early embryo [26]. *Lv*-*elk* is expressed at 10–18 hpf in the skeletogenic mesoderm (Fig. 1a′–e′, arrows). From 20 to 22 hpf, *Lv*-*elk* is expressed faintly in the non-skeletogenic mesoderm but not in the skeletogenic mesoderm (Fig. 1f′–g′, brackets). Beginning in some embryos at 22 hpf and in all embryos by 24 hpf, *Lv*-*elk* is back on in a subset of skeletogenic mesoderm near the tips of the arms and is not detected in the non-skeletogenic mesoderm (Fig. 1h′, arrow). Later, starting in some embryos at 26 hpf and through 32 hpf, *Lv*-*elk* is expressed faintly in 1–2 cells of the apical organ (Fig. 1i′–l′, arrowheads in j′-l′). The late timing of expression of *Lv*-*elk* in the apical organ at pluteus stages suggests that it could have a role in the differentiation of neurons there, similar to its role in the rodent brain. At 32 hpf, many embryos do not show expression of *Lv*-*elk* in the apical organ.

### Transcription factors expressed in or near the ciliary band

**Fig. 2:**
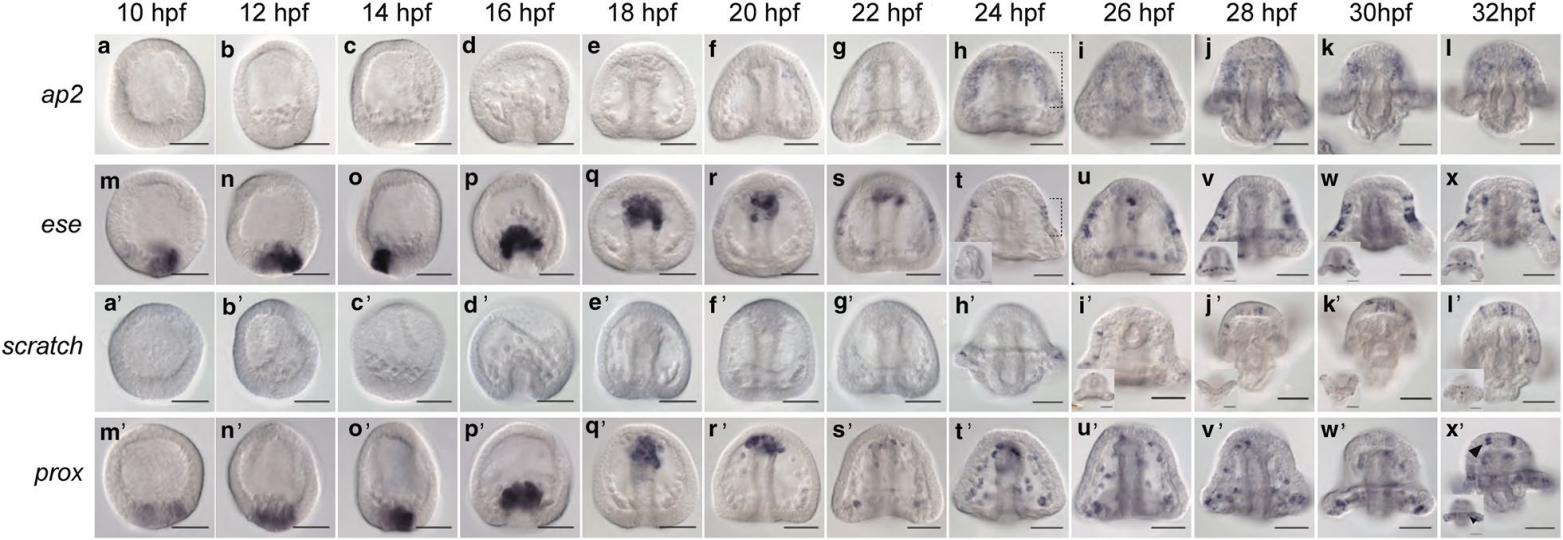
Expression of transcription factors in or near the ciliary band. **a**–**l** *Lv*-*ap2* is expressed starting at 24 hpf in the oral ectoderm near the ciliary band (bracket in **h**) where it remains through 32 hpf. **m**–**x** *Lv*-*ese* is expressed in the non-skeletogenic mesoderm from 10 to 22 hpf. At 22 hpf, *Lv*-*ese* is expressed in the lateral ciliary band (shown in bracket in **t**, inset image shows lateral perspective). It remains in cells of the lateral ciliary band and also turns on in the postoral neurons (shown in inset images in **v**–**x**). **a′**–**l′** *Lv*-*scratch* is expressed beginning at 24 hpf in cells scattered in the ciliary band. Cells expressing *Lv*-*scratch* increase in number through 32 hpf. **m′**–**x′** *Lv*-*prox* is expressed in the non-skeletogenic mesoderm until 32 hpf when it is also expressed throughout the ciliary band (arrow heads in **x′**). Scale bars = 50 μm

AP-2 is a transcription factor that in *Drosophila* is expressed in the developing central nervous system and optic lobes. AP-2 null embryos have brain defects [27–29]. In vertebrates, AP-2 is expressed in neural crest cells and is required for neural crest induction, differentiation, and survival [30–32]. In the sea urchin, *Lv*-*ap2* is expressed starting at 24 hpf in the oral ectoderm in an area encased by the ciliary band (Fig. 2a–l, bracket in h), similar in pattern to expression of *Lv*-*netrin* (Fig. 4h–l). This region of the oral ectoderm in the sea urchin embryo has neurogenic potential, and SoxC-expressing neural progenitors are found there at the time *Lv*-*ap2* is expressed [5]. Perturbations of *Lv*-*ap2* will be necessary to determine what role *Lv*-*ap2* might be having in this region.

Ese is a transcription factor in the Ets family, members of which play roles in neurogenesis in *Drosophila* [33]. Expression of *ese* has been previously reported in a related sea urchin species in the non-skeletogenic mesoderm [34], which is also shown by in situ here in *Lytechinus* (Fig. 2m–s). However, at 22 hpf, *Lv*-*ese* is also expressed in a salt and pepper pattern of cells in the lateral ciliary band (Fig. 2s). By 26 hpf, *Lv*-*ese* expression remains in the lateral ciliary band and extends to the postoral neurons (Fig. 2u–x, bracket in T, inset image in T shows a lateral view). It is possible that *Lv*-*ese* is involved in either specification or differentiation of neural cells in the sea urchin, though it is unclear why *Lv*-*ese* is expressed in only the lateral sides of the ciliary band, and not scattered throughout the entire ciliary band.

Scratch, a member of the snail family of transcription factors, is a pan-neuronal marker in *Drosophila* and is largely confined to the central nervous system of vertebrates [35, 36]. In *Drosophila*, *scratch* promotes neuronal fate as scratch null flies show a significant loss of neurons, while ectopic *scratch* expression leads to additional neurons in the embryo [35]. In mice, *scratch* is expressed largely by postmitotic neurons [36]. In the sea urchin, *Lv*-*scratch* is not detected by in situ hybridization until 24 hpf when it begins in scattered cells throughout the ciliary band in some embryos (Fig. 2a′–h′). As time proceeds, more *Lv*-*scratch* expressing cells are found throughout the ciliary band (Fig. 2i′–l′). The salt and pepper pattern of expression in the ciliary band at pluteus stages is similar to that of the proneural transcription factor *neurogenin* [2]. The restricted and relatively late expression pattern suggests that *Lv*-*scratch* has a function more similar to that in vertebrates.

Prospero or Prox is a homeodomain transcription factor that in vertebrates and in *Drosophila* acts in neural stem cells to regulate the switch from self-renewal to differentiation into postmitotic neurons [37, 38]. In a related sea urchin species, *prox* has been shown to be expressed in non-skeletogenic mesoderm [39, 40]. *Lv*-*prox* is expressed similar to previously reported at 10 hpf in the non-skeletogenic cells that undergo an epithelial-to-mesenchymal transition at the tip of the archenteron at 18–20 hpf (Fig. 2m′–r′). Through 30 hpf, *Lv*-*prox* remains in mesodermal cells (Fig. 2m′–w′). However, by 32 hpf, *Lv*-*prox* is scattered throughout the ciliary band (Fig. 2x′). The late timing of expression in cells that are likely neural suggests that *Lv*-*prox* is not involved in early neural specification. Rather, it could be involved in differentiation or in regulating asymmetric divisions of neuroblasts in the sea urchin.

### Transcription factors expressed in the developing foregut

**Fig. 3:**
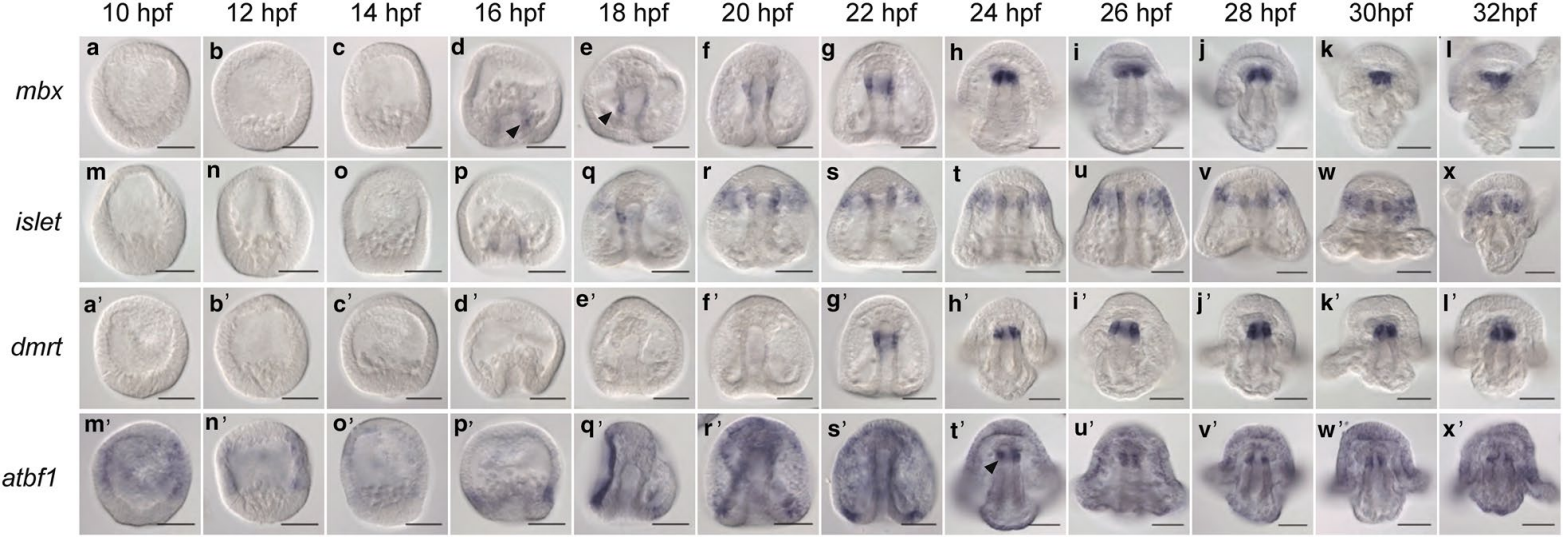
Transcription factors expressed in the foregut. **a**–**l** Expression of *Lv*-*mbx* begins at 16 hpf in the elongating archenteron (arrowhead in **d**, **e**). *Lv*-*mbx* is expressed in the foregut from then through 32 hpf and is also expressed at pluteus stages in the ectoderm near the mouth. **m**–**x** *Lv*-*islet* is expressed in the developing foregut from 16 to 32 hpf. Starting at 18 hpf, *Lv*-*islet* expression is not just in the foregut but in the stripe of ectoderm near the foregut (**q**). **a′**–**l′** *Lv*-*dmrt* is expressed in the foregut from 22 hpf (**g′**) through 32 hpf. **m′**–**x′** *Lv*-*atbf1* is expressed in the ectoderm from 10 to 22 hpf. At 24 hpf, *Lv*-*atbf1* also turns on in the foregut (arrowhead in **t′**), where it remains through 32 hpf. Scale bars = 50 μm

*Diencephalon/mesencephalon homeobox 1 (mbx1)* is expressed in the mouse fore and midbrain regions and is required for proper eye and tectum development in zebrafish [41, 42]. No Mbx ortholog has been found in *Drosophila* [42]. *Lv*-*mbx1* is expressed in a stripe of expression in the developing gut starting at 16 hpf (Fig. 3d, arrowheads in d, e). Once the archenteron has reached its highest point at late gastrula stage, *Lv*-*mbx1* expression is in the foregut and the ectoderm near the developing mouth through 32 hpf (Fig. 3e–l). Thus, if *Lv*-*mbx1* is neural in the sea urchin, it is mostly likely only used in the development of the neurons surrounding the larval mouth. However, functional perturbations of *Lv*-*mbx* are required to determine whether it is playing a role in neurogenesis or if it has a purely endodermal role.

The LIM homeodomain transcription factor, *islet1*, is expressed and required for proper the development of motor neurons of the neural tube, neural crest-derived dorsal root ganglia neurons, and sympathetic neurons [43–45]. *Islet* is also required for the development of *C. intestinalis* palps, which are ectodermal thickenings that give rise to peripheral neurons [46]. Prior to 16 hpf, *Lv*-*islet* expression is not detected by in situ in *Lytechinus* (Fig. 3m–o). *Lv*-*islet* is expressed in some, but not all, embryos beginning at 16 hpf in the developing gut (Fig. 3p). By 18 hpf, *Lv*-*islet* is expressed in a transverse stripe in the anterior portion of the embryo that spans the oral ectoderm and foregut (Fig. 3q). This stripe of expression remains through 32 hpf (Fig. 3r–x). In some embryos at 32 hpf, 1–5 cells in the apical organ also expressed *Lv*-*islet.* It is possible that *Lv*-*islet* is involved in neurogenesis surrounding the larval mouth; however, it is unclear what the function of the ectodermal stripe of expression may be. Perturbation assays will be necessary to determine what role, if any, *Lv*-*islet* is playing in neurogenesis.

Doublesex- and mab-3-related transcription factors (Dmrt family) are perhaps best studied in the context of sexual development. In vertebrates, *C. elegans* and flies, Dmrt family members are involved in sex determination and sexual development [47]. However, members of the Dmrt family are also expressed in the central nervous system and placodes of vertebrate embryos, the neural tube of the tunicate *C. intestinalis,* and have been shown to be required for proper neural development in the *X. laevis* olfactory system [48, 49]. *Lv*-*dmrt* is detected by in situ hybridization beginning at 22 hpf in the foregut, where it remains through pluteus stage (Fig. 3a′–l′). Some embryos begin to express *Lv*-*dmrt* in the foregut beginning at 20 hpf. The timing and placement of *Lv*-*dmrt*, *Lv*-*mbx,* and *Lv*-*islet* expression in the foregut endoderm suggest that perhaps this is an area of active neurogenesis that is required for swallowing behaviors in the embryo.

Atbf1, a zinc finger homeobox transcription factor, has been shown to promote differentiation of neurons by inducing cell cycle arrest [50]. *Atbf1* has been published as being expressed in the ectoderm of a related sea urchin species, *Paracentrotus lividus* [51]. Here we show that *Lv*-*atbf1* shows the same pattern of expression in the ectoderm of *Lytechinus*, but has an additional territory of expression in the foregut. *Lv*-*atbf1* is expressed in the ectoderm from 10 to 18 hpf with a stronger expression in the aboral (non-neural) ectoderm, as reported by Saudemont et al. (Fig. 3m′–q′). At 22 hpf, *Lv*-*atbf1* is also expressed in the ectoderm surrounding blastopore (Fig. 3s′). By 24 hpf, *Lv*-*atbf1* is expressed in the foregut, in a pattern very similar to *Lv*-*dmrt* and *Lv*-*mbx1* (Fig. 3t′ arrowhead). Additional sites of expression of *Lv*-*atbf1* in the *L. variegatus* nervous system include the lateral sides of the ciliary band, apical organ, and the ectodermal ridge where postoral neurons are located (Fig. 3u′-x′).

### Expression of axon guidance molecules

**Fig. 4:**
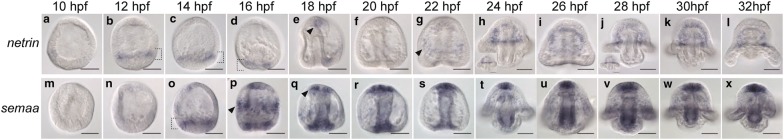
Expression of axon guidance molecules. **a**–**l** *Lv*-*netrin* is expressed from 12 to 16 hpf in a ring in the vegetal plate (brackets in **b**–**d**). *Lv*-*netrin* turns on briefly in the apical organ (arrowhead in **e**). By 22 hpf, *Lv*-*netrin* is expressed in the edges of the oral ectoderm near the ciliary band. **m**–**x** *Lv*-*semaa* is expressed in a ring in the vegetal plate at 14 hpf (bracket in **o**). At 16 hpf, it is also expressed in the ventral ectoderm (arrowhead in **p**) and the apical organ. Through 32 hpf, *Lv*-*semaa* is expressed in the apical organ and the hindgut. Scale bars = 50um

Netrins are secreted laminin-related molecules that have highly conserved roles throughout Metazoa to guide axonal migration [52]. *Lv*-*netrin* is expressed in a bilaterally symmetric pattern in the vegetal plate from 12 to 16 hpf (Fig. 4a–d, brackets in b–d). At 18 hpf, vegetal expression of *Lv*-*netrin* remains in the vegetal plate and is expressed faintly in the apical organ (Fig. 4e, arrowhead). By 22 hpf, *Lv*-*netrin* is expressed in the oral ectoderm in a region not inside the ciliary band itself but bounded by the ciliary band, where it remains through 32 hpf (Fig. 4g–l, arrowhead in G). Axonal tracts have been shown to reside throughout the ciliary band of the sea urchin, but it is unclear what molecules are required for axonal guidance, repulsion, and migration [9]. The expression of *Lv*-*netrin* surrounding the ciliary band suggests that it is used in guidance of axons in the sea urchin embryo.

Semaphorins are a group of secreted and membrane-bound proteins with roles in axon guidance and repulsion and are highly conserved functionally throughout the animal kingdom [53]. *Lv*-*semaphorina (Lv*-*semaa)* is first expressed at 14 hpf when it is detected in the vegetal plate and in the apical organ in some embryos (Fig. 4m–o, bracket in o). By 16 hpf, *Lv*-*semaa* is expressed in the vegetal plate, apical organ domain, and in a band in the ventral ectoderm (Fig. 4p, arrowhead shows ventral ectoderm expression). By 18 hpf, the expression in the ventral band is reduced or off in some embryos and expression remains in the blastopore and apical organ (Fig. 4q, arrowhead shows apical organ expression). Apical and hindgut expression continues through pluteus stage (Fig. 4r–x). It is interesting that *Lv*-*semaa* is expressed at blastula stages, since at this point neural progenitors have just begun to be specified and axonal tracts are not detected. The regional specificity of *Lv*-*semaa* suggests that different regions of the sea urchin nervous system require different axon guidance molecules. Perhaps a diversity of axon guidance molecules is required for regional specificity and connectivity of the nervous system.

### Expression of genes important for neural survival or proliferation

**Fig. 5:**
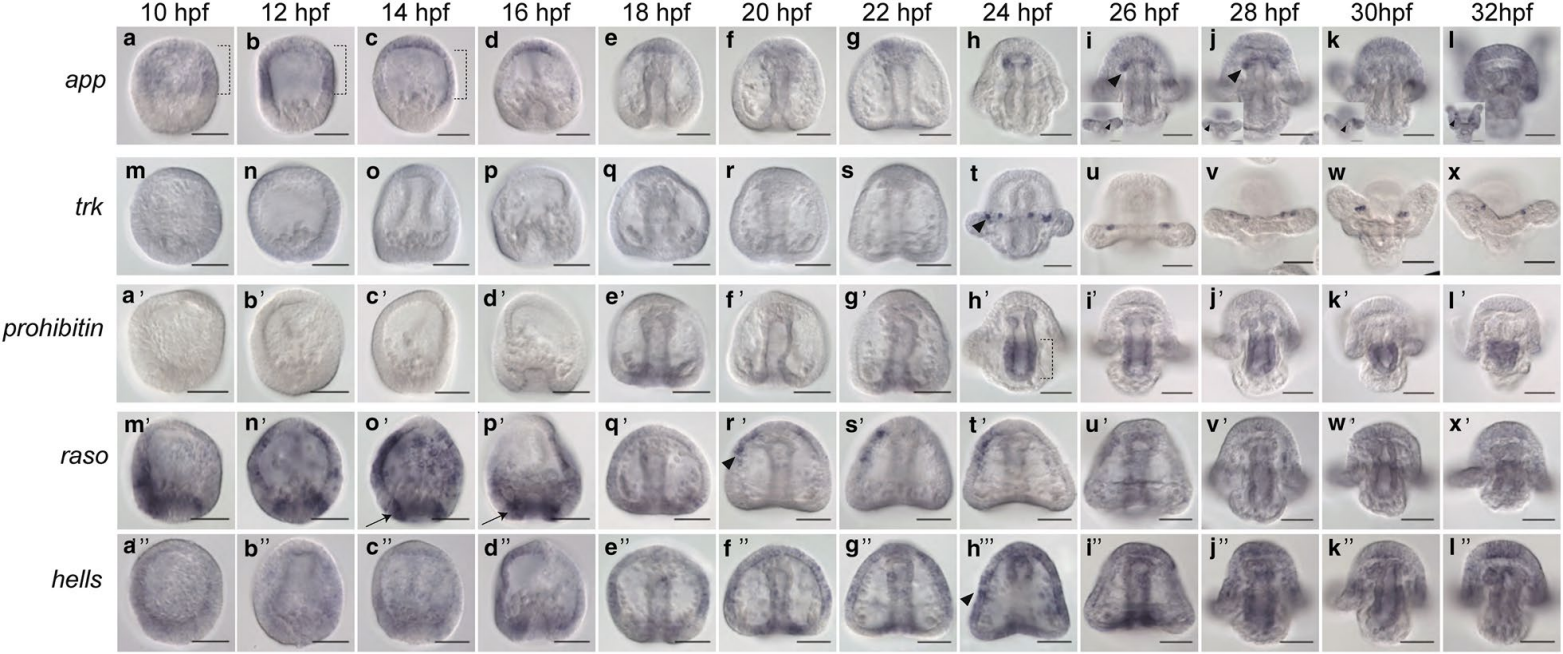
Expression of survival and proliferation genes in the nervous system. **a**–**l** *Lv*-*app* is expressed in the anterior two-thirds of the embryo from 10 to 14 hpf (brackets in **a**–**c**). At 24 hpf, *Lv*-*app* is expressed in the tip of the archenteron, and by 26 hpf, it is expressed in the coelomic pouches (arrowhead in **i**) and in the postoral neurons (inset images in **i**–**l**). **m**–**x** *Lv*-*trk* is not expressed until 24 hpf when it turns on in the postoral neurons (arrowhead in **t**) and remains there through 32 hpf. **a′**–**l′** *Lv*-*prohibitin* is expressed in the mid- and hindgut (bracket in **h′**) beginning at 24 hpf through 32 hpf. **m′**–**x′** *Lv*-*rasO* is expressed in the ectoderm and vegetal plate at 10 hpf. By 14 hpf, *Lv*-*rasO* remains in the ectoderm and is in a band of expression surrounding the vegetal plate (arrows in **o′**, **p′**). By 20 hpf, *Lv*-*rasO* is expressed in one or both lateral sides of ciliary band (arrowhead in **r′**). By 22 hpf, *Lv*-*rasO* expression is on a single side of the lateral ciliary band. Expression then diminishes by 28 hpf. **a″**–**l″** *Lv*-*hells* is expressed in the ciliary band (arrowhead in **h″**) as well as the apical organ and hindgut. Scale bars = 50 μm

APP or amyloid-β precursor protein is a transmembrane protein shown to be important for the formation and transmission of synapses of neurons in culture and crucial to the pathology of Alzheimer’s disease [54]. *Lv*-*app* is expressed in the ectoderm of the anterior two-thirds of the embryo until 16 hpf when its expression is detected lightly in the animal pole domain in some, but not all embryos (Fig. 5a–g, brackets in a–c). At 24 hpf, *Lv*-*app* is expressed in the top of the foregut and by 26 hpf it is expressed in the coelomic pouches (Fig. 5h, i, arrowhead in i). At this time point, some embryos begin to express *Lv*-*app* in the postoral neurons (arrowhead in inset image of Fig. 5i). At 28 hpf, *Lv*-*app* remains faintly in the pouches and is expressed in the postoral neurons (Fig. 5j, arrowheads). From 30 to 32 hpf, *Lv*-*app* is expressed in the postoral neurons but is no longer detected in the coelomic pouches (Fig. 5k–l, arrowheads in inset images of k, l show postoral neurons). The late expression of *Lv*-*app* in the postoral neurons, several hours after they have been specified, suggests that it is involved in survival, differentiation, or maintenance of these neurons. However, the function of *Lv*-*app* in other areas of the embryo, particularly in non-neural tissues such as the mesodermal coelomic pouches, is unclear.

Neurotrophic receptor tyrosine kinase (TRK) genes are essential for neuron survival in a subset of the vertebrate central and peripheral nervous systems (reviewed in [55]). *Lv*-*trk* is first detected in the postoral neurons in some, but not a majority, of embryos by in situ hybridization at 22 hpf (Fig. 5m–t). By 24hpf, *Lv*-*trk* is expressed in the postoral neurons where it remains through 32 hpf (Fig. 5t–x, arrowhead in t). This regional expression of *Lv*-*trk* in only the postoral neurons suggests that different survival genes are required for different subtypes of neurons in the sea urchin.

Prohibitin is a protein that has been shown to confer neuron survival by reducing the production of free radicals in mitochondria [56]. It has also been shown to play a specification role in *Xenopus* neural crest [57]. In *Lytechinus*, expression of *Lv*-*prohibitin* is not detected by in situ hybridization from 10 to 16 hpf (Fig. 5a′–d′). Signal begins to accumulate for *Lv*-*prohibitin* in the hindgut starting at 18 hpf (Fig. 5e′). Overtime, *Lv*-*prohibitin* continues to be expressed in the mid and hindgut (Fig. 5f′–l′, bracket in h′). Neurons of the pyloric and anal sphincters originate in those regions so it is possible that *Lv*-*prohibitin* is involved in neurogenesis there, though it is also possible that *prohibitin* does not perform a neural role in the sea urchin.

Ras signaling has been extensively studied in the context of neurogenesis. Ras signaling has been shown to be used to select cell fates in the mouse brain [58, 59]. Ras proteins are also involved in specification of glial cells in the embryonic brain [60]. *Ras family orphan* or *Lv*-*rasO* is expressed from 10 to 16 hpf in a ring in the vegetal plate surrounding the future site of the blastopore and diffusely in the ectoderm (Fig. 5m′–p′, arrows in o′ and p′ show blastoporal expression). At 18 hpf, it is expressed in and around the developing blastopore as well as diffusely in the ectoderm (Fig. 5q′). At 20 hpf, *Lv*-*rasO* is expressed in one or both lateral sides of the ciliary band (Fig. 5r′, arrowhead). By 22 hpf, *Lv*-*rasO* expression is in the ciliary band in a very peculiar expression pattern. In the ciliary band, *Lv*-*rasO* is asymmetric- only being expressed in one side of the embryo in the lateral ciliary band (Fig. 5s′). This particular asymmetry within the ciliary band is not seen in any other neural genes in this screening nor in any published neural expression patterns in the sea urchin and the purpose of this asymmetric expression is unclear. Starting at 26 hpf through 32hpf, *Lv*-*raso* expression is largely diminished (Fig. 5t′–x′).

Lymphoid-specific helicase isoform 1 (Hells) is expressed in lymphoid precursor cells and has no reported role in neurogenesis [61]. However, *Lv*-*hells* is expressed in neural territories in the sea urchin. *Lv*-*hells* is expressed lightly from 10 to 16 hpf scattered in the ectoderm (Fig. 5a″–d″). By 18 hpf, *Lv*-*hells* is expressed in the ciliary band and blastopore and beginning at 24 hpf in the ciliary band, hindgut/blastopore and foregut (Fig. 5e″–l″, arrowhead in H′ shows ciliary band expression). Members of the helicase family regulate processes such as control of transcription and DNA repair and perhaps are providing the same role in neural territories of the sea urchin [61, 62].

### Expression of neurotransmitter-related genes

**Fig. 6:**
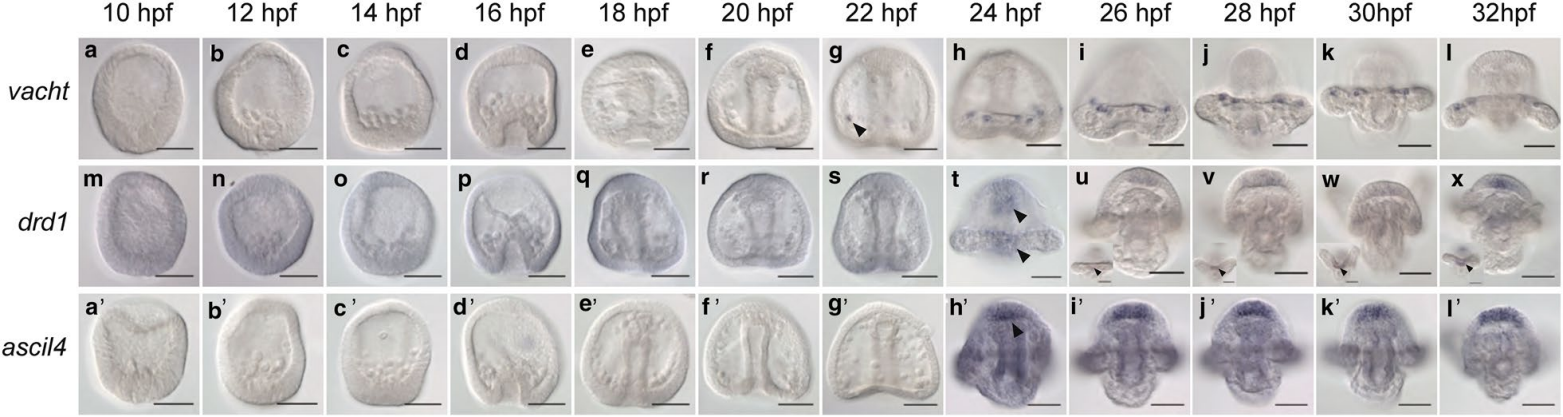
Expression of neurotransmitter-related genes. **a**–**l** *Lv*-*vacht* is expressed beginning in some embryos at 22 hpf in the postoral neurons (arrowhead in **g**) where it remains through 32 hpf. **m**–**x** *Lv*-*drd1* is expressed starting at 24 hpf in the ectoderm near the stomodeum and near the postoral neurons (arrowheads in **t**), where it remains through 32 hpf. **a′**–**l′** *Lv*-*asicl4* is expressed beginning at 24 hpf in the developing apical organ (arrowhead in **h′**) through 32 hpf. Scale bars = 50 μm

Vacht is a transporter protein responsible for loading acetylcholine into secretory vesicles in neurons [63, 64]. In a pattern very similar to *Lv*-*chat* [2], *Lv*-*vacht* is expressed in the postoral neurons. It is not expressed from 10 to 18 hpf (Fig. 6a–e) and begins to be expressed in some embryos at 20 hpf where it remains through 32hpf (Fig. 6g–l, arrowhead in g).

Dopamine receptor D1, or *Lv*-*drd1*, is first detected in some embryos beginning at 24 hpf in the anterior ectoderm near the mouth and in a patch in the posterior ciliary band (Fig. 6m–t, arrowheads in t). It remains expressed in those areas faintly until 32 hpf (Fig. 6u–x, arrowheads in inset images show posterior ciliary band expression). It makes sense that expression of *Lv*-*drd1* is found near the mouth and postoral neurons since expression of *Lv*-*th*, expressed by dopaminergic neurons, has been reported to be expressed in those regions [2].

Members of the acid sensing ion channel (Asic) family are ion channels found throughout the vertebrate nervous system that help convert chemical signals to electrical current [65]. *Asic* genes are conserved throughout deuterostomes but are not found in protostome animals [65]. In *Lytechinus*, *Lv*-*asicl4* shows no expression from 10 to 22 hpf (Fig. 6a′–g′). Expression is detected by in situ beginning at 24 hpf in the apical organ where it remains through 32 hpf (Fig. 6h′–l′, arrowhead in h′). *Lv*-*asicl4* is likely involved in the function of neurons in the apical organ, since its expression begins after the serotonergic neurons have differentiated, as marked by expression of serotonin.

## Discussion

### The sea urchin embryonic nervous system is subdivided into distinct regulatory states

Over the past several years, we have gained a better understanding of when and where neurons are specified and differentiate in the sea urchin embryo. That provides the opportunity to look at genes associated with neurogenesis elsewhere in the animal kingdom and ask which of these are associated with neural development in the sea urchin. When and where do these genes appear in the sea urchin? Which are exclusively expressed in neural territories and which are expressed in other locations in the embryo? If these genes are neural in the sea urchin, to which regulatory state do they likely belong?

To this end, the in situ hybridization data shown here allow us to begin addressing those questions. The ciliary band, the apical organ, the foregut endoderm, the hindgut endoderm, and the oral ectoderm each harbor neurons in the larva [2, 5, 10, 12, 66, 67]. Most genes in this survey are expressed in one or more of these territories in a manner that suggests a neural association. However, expression within an area of the nervous system alone does not mean that a gene has a neural function. It is entirely possible that some of these genes do not play a role in neurogenesis. However, if a gene has been shown to be neurogenic in multiple organisms of different lineages (including protostome and vertebrate models) and is expressed in developing neural territories in the sea urchin, then it is likely to have a neurogenic role there. This is particularly true of the genes that are expressed in a salt and pepper pattern within the nervous system (such as *Lv*-*scratch, Lv*-*ese* and *Lv*-*prox,* Fig. 2), which is a classic characteristic of neural patterning. We believe that all of the gene expression patterns shown here play some role in neurogenesis, be it specification of neurons, axon guidance, or neural survival. Careful co-expression analysis followed by perturbations will be required to determine what role, if any, each of these genes has during neurogenesis in the sea urchin.

**Fig. 7:**
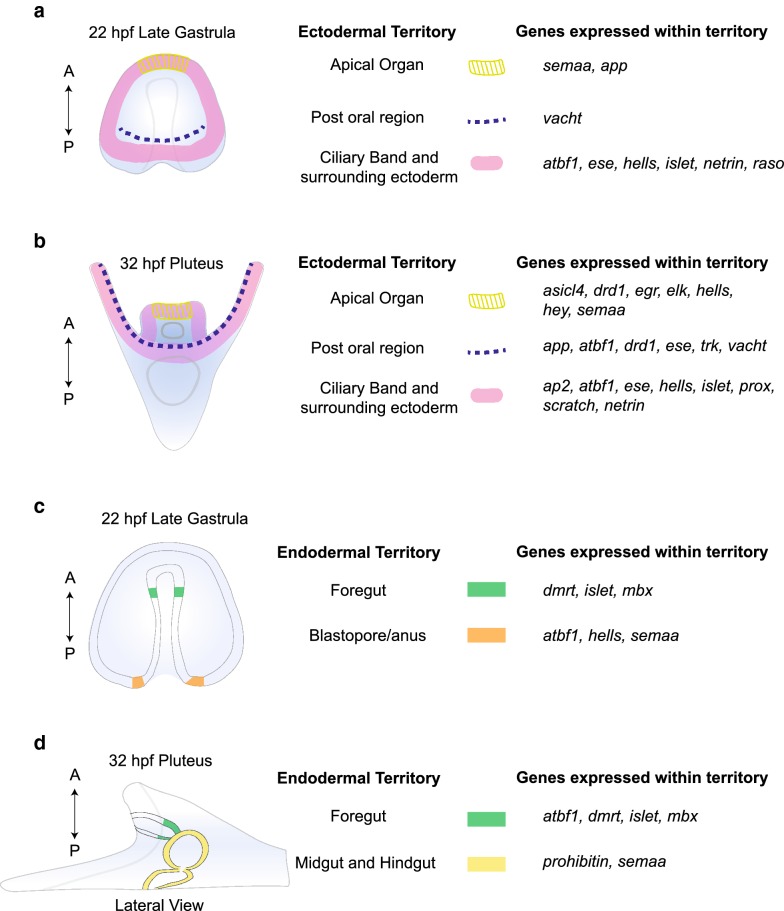
Schematic of genes expressed in different regions of the ectoderm and endoderm at late gastrula and pluteus larva stages. **a**, **b** Schematics show 3 ectodermal territories (apical organ, ciliary band, and postoral region) and the list of genes from this study expressed in those regions at two time points (22 hpf and 32 hpf). Expression within a region does not mean that the genes are necessarily co-expressed with one another. **c**, **d** Schematics show endodermal territories where genes from this study are expressed at late gastrula and pluteus stages. Expression within a region does not mean that the genes are necessarily co-expressed. *A* anterior, *P* posterior

Nevertheless, the data presented here suggest that these territories each have their own unique gene expression profile, and therefore, each represents a distinct regulatory state (Fig. 7, Table 1). Furthermore, gene expression data here suggest that the ciliary band itself can be subdivided further into other regulatory states as suggested by Barsi and colleagues [68]. For example, the transcription factor *Lv*-*ese* is expressed in cells only in the lateral sides and posterior ciliary while other transcription factors such as *Lv*-*scratch* and *Lv*-*prox* are expressed in cells scattered throughout the ciliary band. This suggests neural cells in different regions of the ciliary band express different combinations of transcription factors and perhaps carry out different functions and have different trajectories. At the same time, a number of the genes presented in this study are expressed in patterns that suggest additional territories of expression outside the nervous system.

**Table 1:**
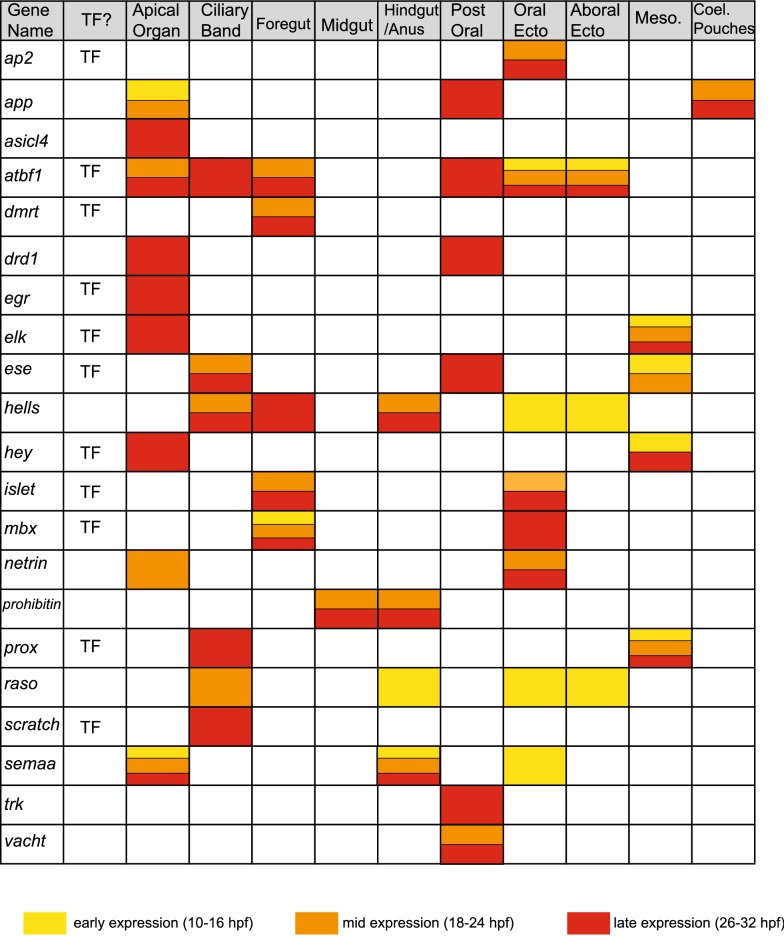
Areas and timing of expression

Rows indicate genes, and columns indicate areas of the embryo. White/blank indicates no expression detected in that area. Colors indicate timing of when the gene is expressed in that area. Yellow: early expression (at some point between 10 and 16 hpf). Orange: mid-timing of expression (at some point between 18 and 24 hpf). Red: late expression (at some point between 26 and 32 hpf)

*TF* transcription factor, *Postoral* postoral neurons or ectoderm surrounding the postoral neurons, *Oral Ecto* oral ectoderm, *Aboral Ecto* aboral ectoderm, *Meso*. mesodermal cells/tissues, *Coel. Pouches* coelomic pouches

The data provided here are not intended to be a complete list of molecular players expressed in the embryonic nervous system. It is highly likely that there are many other genes expressed in the sea urchin nervous system but were not identified in this screening because they turn on earlier than 10 hpf or later than 32 hpf. Furthermore, there are likely other genes that are neurogenic in the sea urchin but were not found in this screening because they do not fall under gene ontology (GO) term categories related to nervous system development. While gathering information about regulatory states in an embryo is necessary for building a developmental gene regulatory network, it cannot tell us about interactions between genes and which molecular players are essential for development. What it can do is provide a guide for future experiments designed to determine how neurogenesis occurs in each of the several regulatory regions. It is clear that each region of the embryonic nervous system is distinct in the neural genes expressed so each must be considered separately in terms of specification trajectory, timing, and differentiation.

While understanding a neurogenic GRN for a basal deuterostome will be informative from an evolutionary perspective, it can also allow for comparisons between the embryonic and adult (postmetamorphic) nervous systems. It is believed that the larval nervous system is completely lost during metamorphosis and that the adult nervous system is formed de novo [69]. This leaves the question of whether the same genes are required for the formation of the adult nervous system and whether the same or a modified version of the embryonic GRN is deployed after metamorphosis for the formation of the adult nervous system.

### Expression of genes in multiple regions of the embryo

Several genes in this study are expressed in multiple territories in the embryo (Table 1). These include the transcription factors *Lv*-*ese, Lv*-*prox*, *Lv*-*islet, Lv*-*atbf1*, *Lv*-*hey,* and *Lv*-*elk* as well as the axon guidance gene *Lv*-*semaa.* Some of these are expressed in multiple neural territories while others are expressed in neural domains and in mesodermal cells (Table 1). This underscores a limitation of using purely quantitative methods including quantitative PCR and RNAseq alone to build gene regulatory networks. These methods, while extremely sensitive and quantitative, do not give a spatial picture of where in the embryo genes are affected by perturbations. For this, one needs in whole mount expression assays.

Spatiotemporal expression is one of the most valuable components necessary for building GRNs. The spatiotemporal pattern of expression of the genes in this analysis indicates that there are significant differences of expression in the three primary neurogenic territories. This provides a framework for perturbation studies to determine what genes are more upstream in these neural territories and which genes are involved in downstream processes of neural development. The obvious logic of GRNs is that a gene expressed early is far more likely to be nearer to the top of a GRN than a gene that is first expressed later in development. Similarly, genes that are expressed with a close temporal sequence to another gene’s expression may provide hints of direct relationships. Bolouri and Davidson [70] showed for *S. purpuratus* that it took on average 3 h for the RNA of one gene to be expressed, processed, translated into a functional protein before it could activate the next downstream gene. In *Lytechinus,* that “step time” is likely shorter since *Lytechinus* develops in sea water that is about 8–10 °C warmer than for *S. purpuratus*.

It is possible that genes expressed both in mesodermal tissues and in neurogenic territories are performing similar roles in both regions. Notably, both secondary mesoderm and neurogenesis have been shown to be dependent on Delta/Notch signaling in the sea urchin [2, 10, 15, 71–74]. It could be that the genes expressed in both mesoderm and neural territories are dependent on or are regulators of Notch signaling in both tissue types.

Dynamic patterns of expression are also revealed by this analysis. For some genes, expression appears to be broad and later narrows to just a few cells. Expression of other genes begins in one cell at a time in a salt and pepper pattern. Expression of some genes occurs only during a limited timeframe meaning that as a GRN state switches, it must be turned on and then later turned off. The timing of that temporary expression could occur at a specific time for an individual cell or roughly simultaneously for all cells within a territory. Each of these patterns offers early hints toward the role of that protein in establishing a nervous system in the larva.

A major difficulty with this kind of analysis is the inability to track a single cell over time. Since the analysis reports expression of a field of cells in each territory, the analysis quickly becomes complicated if, as is likely, neurogenesis starts with a few cells and then additional cells initiate neurogenesis. Given that likelihood, it is very difficult to establish, a priori, the sequence of gene expression. For that reason, perturbations are absolutely necessary to gain a reasonable understanding of expression sequences and connections in forming a GRN. This analysis thus is a series of snapshots, and one interpretation, lacking other information, is that if a gene is expressed at one stage and then expressed in successive stages, the gene is continuously expressed. However, one should be aware that other possibilities exist, e.g., the gene is transiently activated in neurons which are born at different times during development. Because this analysis did not follow single cells through time, either outcome is possible. Injection of a recombinant BAC for each gene would allow a time lapse expression analysis (via live imaging) and would help resolve the present ambiguity. To produce recombinant BACs to a large number of genes is a cumbersome task but may be important for resolving some of the issues of GRN assembly in future experiments. With all of these caveats, however, it is still extremely useful to obtain a dataset such as that contained in this analysis before proceeding to assemble a GRN reflecting the generation of a nervous system.

## Conclusions

The results of this in situ hybridization screening provide a map of the regulatory states present in the sea urchin embryo during neurogenesis. The findings presented in this paper will allow for a dissection of the neurogenic gene regulatory network in a basal deuterostome. At this point, we can only speculate about the functions of these genes during development based on findings in other systems. With these data in hand, the next logical step would be to begin using functional perturbation assays to determine the upstream and downstream relationships of these genes and the consequences these genes have on development of the nervous system in the sea urchin. Comparing the results of these functional studies to what is known in other species will provide a looking glass into how evolution shaped neurogenesis in deuterostomes.

## Methods

### Adult animals and embryo culture

Adult *L. variegatus* sea urchins were collected from the Duke University Marine Lab (Beaufort, NC, USA) or Pelagic Corp. (Sugarloaf Key, FL, USA). Gametes were harvested by injection of 0.5 M KCl into adult sea urchins. Embryos were cultured at 23 °C in filtered artificial seawater (ASW).

### Cloning and in situ hybridization

Cloning of full length or partial coding sequences for all genes in this study was carried out by designing primers against a transcriptome data set and cloned into pGEM T-easy vector (Promega). PCR was carried out with High Fidelity Phusion Master Mix (NEB). Accession numbers are shown in Table 2. Additional candidates that, in our hands, did not produce a discernable expression pattern between 10 and 32 hpf by in situ hybridization are listed in Table 3. Whole mount in situ hybridization (ISH) was performed using RNA probes labeled with Digoxigenin-11-UTP (Roche). Large cultures of embryos were fixed in a time course for analysis. For each gene, two different sets of embryos were analyzed for each time point. Embryos were fixed by gentle rocking overnight at 4 °C in 4% paraformaldehyde made in filtered artificial sea water (FASW), washed with FASW at room temperature, and stored in methanol at − 20 °C. RNA probes were synthesized in vitro and hybridized at 65 °C. Probes were visualized using AP-conjugated anti-DIG antibody (1:1500, Roche [Indianapolis, IN, United States]). Color was developed using NBT/BCIP (Roche). For each antisense probe, a sense probe was also tested and shown to show no expression pattern or background signal only (Additional file 1: Figs. S1–S6).

**Table 2:** Plasmids deposited in GenBank

Plasmid	Fragment	Accession
Ap2	Partial CDS	MK015667
App	Partial CDS	MH996686
AsicL4	Partial CDS	MH996683
Atbf1	Partial CDS	MK012222
Dmrt	Partial CDS	MK012223
Drd1	Partial CDS	MH996687
Egr	Partial CDS	MK015664
Elk	Partial CDS	MK029662
Hells	Partial CDS	MK015665
Hey	Full CDS	MK012227
Islet	Partial CDS	MK012226
Mbx	Partial CDS	MK012221
Netrin	Partial CDS	MK015666
Prohibitin	Full CDS	MK015662
RasO	Full CDS	MK015663
Scratch	Partial CDS	MK012224
SemaA	Partial CDS	MK012225
TRK	Partial CDS	MK029664
Vacht	Partial CDS	MK029665

“Partial CDS” means for that gene’s in situ riboprobe, a partial region of the coding sequence was used. “Full CDS” means that the in situ riboprobe was synthesized from the entire coding sequence

**Table 3:** List of candidate genes screened

Gene name	TF?
*arnt*	
*ato*	TF
*bace1*	
*clock*	TF
*drd2*	
*gad*	
*gbx*	TF
*glia*	TF
*gnmt2*	
*grb2*	
*id*	TF
*homer*	
*ldla/gpcr1*	
*machrm5*	
*mec2*	
*nel1l*	
*neurotrophin*	
*p75ntr*	
*pou4/brn3*	TF
*rhop2*	
*semaB*	
*sim*	TF

These genes produced no discernable expression pattern between 10 and 32 hpf in our hands

*TF* indicates genes that act as transcription factors

## Additional file

**Additional file 1.Figure S1:** Sense probes of transcription factors expressed in the apical organ. *In situ* hybridizations show expression patterns for the sense and antisense probes for *egr, hey,* and *elk.* For each gene, *in situ* hybridization with sense probes was done side by side with antisense probes on embryos from the same time point and was left in color solution for the same amount of time. **Figure S2:** Sense probes of transcription factors expressed in or near the ciliary band. *In situ* hybridizations show expression patterns for the sense and antisense probes for *ap2, ese, scratch,* and *prox.* For each gene, *in situ* hybridization with sense probes was done side by side with antisense probes on embryos from the same time point and was left in color solution for the same amount of time. **Figure S3:** Sense probes of transcription factors expressed in the foregut. *In situ* hybridizations show expression patterns for the sense and antisense probes for *mbx, islet, dmrt,* and *atbf1.* For each gene, *in situ* hybridization with sense probes was done side by side with antisense probes on embryos from the same time point and was left in color solution for the same amount of time. **Figure S4:** Sense probes of axon guidance molecules. *In situ* hybridizations show expression patterns for the sense and antisense probes for *netrin* and *semaa.* For each gene, *in situ* hybridization with sense probes was done side by side with antisense probes on embryos from the same time point and was left in color solution for the same amount of time. **Figure S5:** Sense probes of genes involved in neural survival or proliferation in other species. In situ hybridizations show expression patterns for the sense and antisense probes for *app, trk, prohibitin, raso,* and *hells.* For each gene, *in situ* hybridization with sense probes was done side by side with antisense probes on embryos from the same time point and was left in color solution for the same amount of time. **Figure S6:** Sense probes of neurotransmitter-related genes. *In situ* hybridizations show expression patterns for the sense and antisense probes for *vacht, drd1,* and *asicl4.* For each gene, *in situ* hybridization with sense probes was done side by side with antisense probes on embryos from the same time point and was left in color solution for the same amount of time.
