# Supplementary material for: Spatial and temporal patterns of gene expression during neurogenesis in the sea urchin Lytechinus variegatus
Source: EvoDevo. 2019 Feb 12;10:2. doi: 10.1186/s13227-019-0115-8 (PMC6371548; doi:10.1186/s13227-019-0115-8)

Additional Figure S1: Sense probes of transcription factors expressed in the apical organ

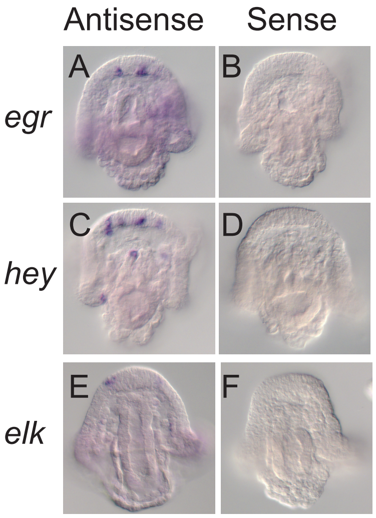

Additional Figure S2: Sense probes of transcription factors expressed in or near the ciliary band

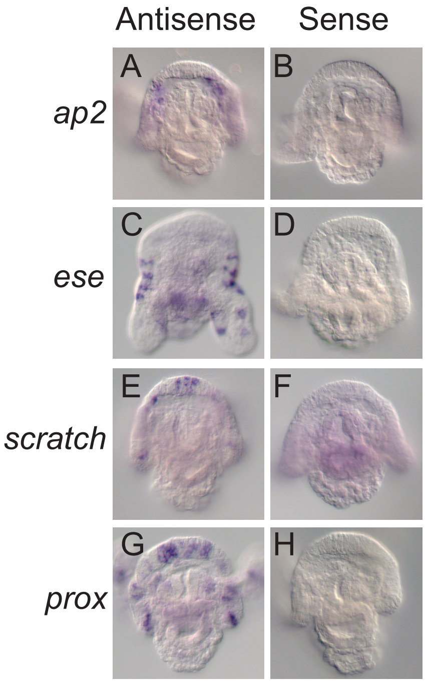

Additional Figure S3: Sense probes of transcription factors expressed in the foregut

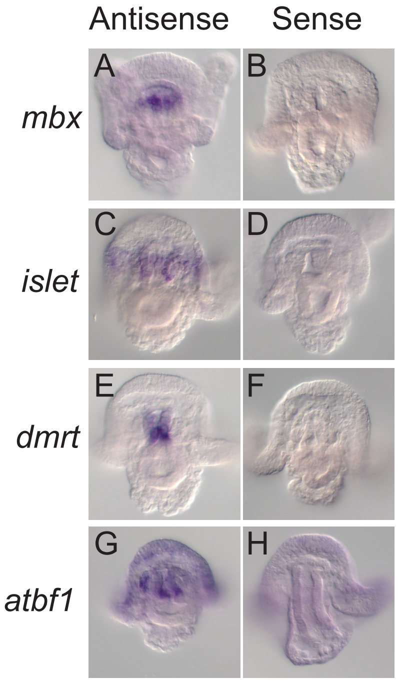

Additional Figure S4: Sense probe expression of axon guidance molecules

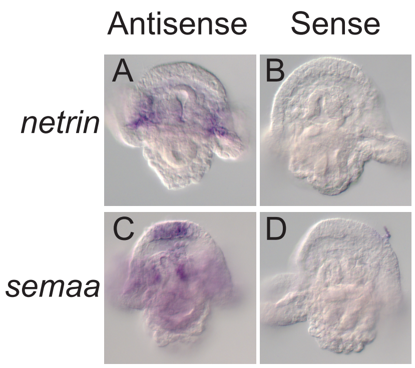

Additional Figure S5: Sense probes of genes involved in neural survival or proliferation

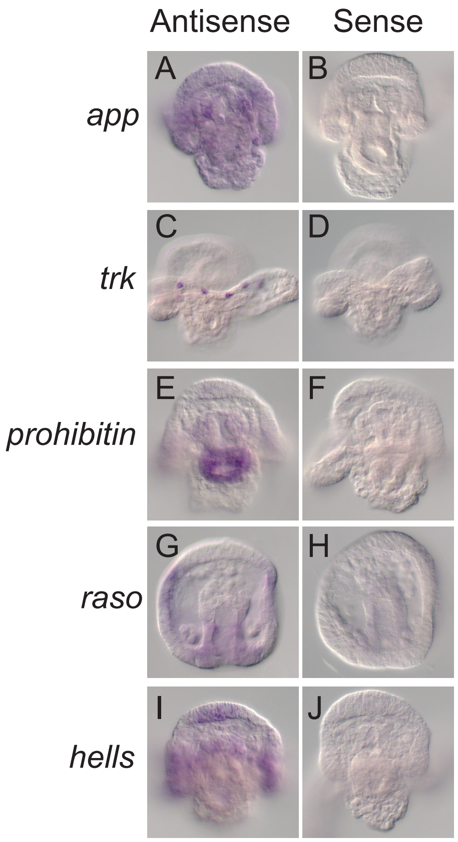

Additional Figure S6: Sense probes of neurotransmitter related genes

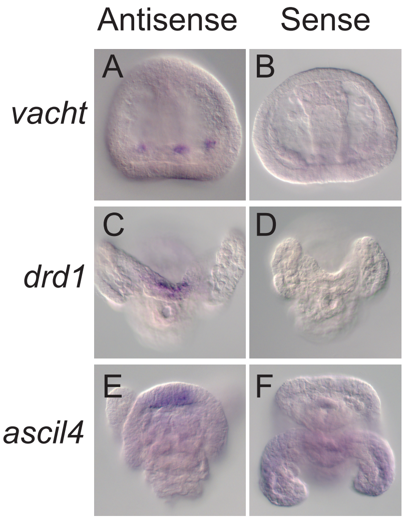

Supplement: Supplementary file 1 — Additional file 1.Figure S1: Sense probes of transcription factors expressed in the apical organ. In situ hybridizations show expression patterns for the sense and antisense probes for egr, hey, and elk. For each gene, in situ hybridization with sense probes was done side by side with antisense probes on embryos from the same time point and was left in color solution for the same amount of time. Figure S2: Sense probes of transcription factors expressed in or near the ciliary band. In situ hybridizations show expression patterns for the sense and antisense probes for ap2, ese, scratch, and prox. For each gene, in situ hybridization with sense probes was done side by side with antisense probes on embryos from the same time point and was left in color solution for the same amount of time. Figure S3: Sense probes of transcription factors expressed in the foregut. In situ hybridizations show expression patterns for the sense and antisense probes for mbx, islet, dmrt, and atbf1. For each gene, in situ hybridization with sense probes was done side by side with antisense probes on embryos from the same time point and was left in color solution for the same amount of time. Figure S4: Sense probes of axon guidance molecules. In situ hybridizations show expression patterns for the sense and antisense probes for netrin and semaa. For each gene, in situ hybridization with sense probes was done side by side with antisense probes on embryos from the same time point and was left in color solution for the same amount of time. Figure S5: Sense probes of genes involved in neural survival or proliferation in other species. In situ hybridizations show expression patterns for the sense and antisense probes for app, trk, prohibitin, raso, and hells. For each gene, in situ hybridization with sense probes was done side by side with antisense probes on embryos from the same time point and was left in color solution for the same amount of time. Figure S6: Sense probes of neurotransmitter-rel [file 13227_2019_115_MOESM1_ESM.pdf]
